# Cost-utility analysis of adjuvant trastuzumab therapy for HER2-positive early-stage breast cancer in the Philippines

**DOI:** 10.1186/s12913-019-4715-8

**Published:** 2019-11-21

**Authors:** Anne Julienne Genuino, Usa Chaikledkaew, Anna Melissa Guerrero, Thanyanan Reungwetwattana, Ammarin Thakkinstian

**Affiliations:** 1 Mahidol University Health Technology Assessment (MUHTA) Graduate Program, Bangkok, Thailand; 2 Pharmaceutical Division, Department of Health Philippines, Manila, Philippines; 3 Social and Administrative Pharmacy Excellence Research (SAPER) Unit, Department of Pharmacy, Faculty of Pharmacy, Mahidol University, 447 Sri-Ayudhaya Rd., Phayathai, Ratchathewi, Bangkok, 10400 Thailand; 4 Division of Medical Oncology, Department of Medicine, Faculty of Medicine Ramathibodi Hospital, Mahidol University, Bangkok, Thailand; 5 Section for Clinical Epidemiology and Biostatistics, Faculty of Medicine Ramathibodi Hospital, Bangkok, Thailand

**Keywords:** Adjuvant trastuzumab, Cost-utility analysis, HER2-positive, Breast cancer, Cost-effectiveness, Philippines

## Abstract

**Background:**

Breast cancer is the leading malignancy among Filipino women, with about 23.50% of cases characterized by human epidermal growth factor receptor-2 (HER2) overexpression. Trastuzumab, in addition to standard chemotherapy, is currently recommended as primary treatment for HER2-positive early-stage breast cancer (EBC) in the adjuvant settings, and has been listed in the Philippine National Formulary (PNF) since 2008, but with no current evidence yet on its value for money, to date. Hence, despite several policy enablers, its accessibility remains to be limited in the Philippines. We performed an economic evaluation to assess the cost-effectiveness and budget impact of adjuvant trastuzumab therapy for HER2-positive EBC in the Philippines, using healthcare system and societal perspectives, in aid of guiding coverage decisions.

**Methods:**

A Markov model-based cost-utility and budget impact analyses were conducted to estimate the total costs incurred and outcomes gained in using 1 year of adjuvant trastuzumab added to standard chemotherapy versus standard chemotherapy alone, over a lifetime horizon. We discounted both costs and outcomes at 3.5% per annum. Parameters were estimated using country survival data, systematic review and meta-analysis of the relative treatment effect, local and international cost data, and published utility data. Univariate and probabilistic sensitivity analyses were used to account for parameter uncertainty.

**Results:**

Trastuzumab therapy was dominated with an incremental cost-effectiveness ratio (ICER) at PHP 453,505 per QALY gained from a healthcare system perspective or PHP 458,686 per QALY gained from a societal perspective, with 10% cost-effectiveness probability at the country cost-effectiveness threshold of PHP 120,000 per QALY gained. National implementation will cost an additional amount of PHP 13,909 million in year one alone, plus about PHP 2000 to 3000 million annually for the succeeding fiscal years.

**Conclusion:**

At its current cost, 1 year of adjuvant trastuzumab therapy compared to standard chemotherapy alone for HER2-positive EBC does not represent value for money in the Philippines. Its current cost will have to significantly lower down by one-half to achieve cost-effectiveness.

## Background

Breast cancer is currently the globally leading malignancy among women with about 1.7 million diagnosed cases and 521,907 deaths as estimated by the World Health Organization (WHO) in 2012 [1]. In the Philippines, it is recognized as the most prevalent cancer among women and in both sexes, as well as the most common cause of cancer deaths among women [2]. About 15 to 20% of breast cancers overexpresses human epidermal growth factor receptor-2 (HER2) [3] – a clinically important subtype of breast cancer that is associated with an aggressive disease phenotype and shortened survival outcomes [4], resulting in poorer prognosis compared to other subtypes. Two large studies of breast cancer data in the United States [5] [6] reported, however, that women of Asian descent were more likely to have HER2-positive tumours than Caucasian women suggesting possible racial differences. In the Philippines, the Department of Health (DOH) Breast Cancer Control Program reported a HER2-positivity rate of 23.17%, with an estimated 80% of them at the early stage [7, 8].

The discovery of revolutionary therapies such as trastuzumab, the first monoclonal antibody to specifically target HER2, has changed the course of treatment and improved the prognosis of affected breast cancer patients. Several key pivotal trials have demonstrated its relative treatment efficacy versus standard chemotherapy alone, in improving the disease-free and overall survival of HER2-positive early-stage breast cancer (EBC) patients [9–16]. These same trials though have reported an associated increased risk for cardiotoxic effects such as congestive health failure (CHF) and left ventricular ejection fraction (LVEF) decline [9–16].

Both global [17, 18] and national clinical guidelines [19] currently recommend the administration of trastuzumab with chemotherapy regimens as primary treatment for HER2-positive EBC in adjuvant settings. It has been listed in the Philippine National Formulary (PNF) since 2008, but with no current evidence yet on its value for money to date. Hence, despite several policy enablers, its accessibility remains to be limited as the current government insurance case rate for breast cancer does not cover the treatment for HER2-positive type; public hospitals cannot afford to procure and make it available in their facilities due to its high cost; and, while it has been recently included in the list of subsidized medicines under the DOH Breast Cancer Medicine Access Program (DOH BCMAP) under a negotiated reduced price for national hospitals, the access sites and medicine stocks are limited.

Assessing cost-effectiveness is critical in establishing equitable trade-off decisions between the sustainable access to such effective health technology that can improve survival and the limited health budget, especially for resource-constrained countries such as the Philippines. While many published economic evaluation (EE) studies have been previously conducted to assess its cost-effectiveness, all were conducted in settings that are not comparable to a lower-middle income country (LMIC) like the Philippines, as they were all from upper-middle and high-income countries [20–35]. Therefore, we conducted this economic evaluation to assess the cost-effectiveness and budget impact of adjuvant trastuzumab therapy compared to chemotherapy regimen alone for patients with HER2-positive EBC in the Philippines, to guide coverage decisions.

## Methods

We conducted a cost-utility analysis (CUA) using decision analytic Markov model to calculate and compare the costs and utilities of using 1 year of adjuvant trastuzumab combined with standard chemotherapy (i.e., doxorubicin 60 mg/m^2^ on day 1 plus cyclophosphamide 600 mg/m^2^ on day 1 every 3 weeks for 4 cycles followed by docetaxel 100 mg/m^2^ on day 1 plus trastuzumab 8 mg/kg initial dose then 6 mg/kg on day 1 every 3 weeks for 4 cycles then trastuzumab 6 mg/kg on Day 1 every 3 weeks for 14 cycles) versus chemotherapy alone (i.e., same chemotherapy minus trastuzumab) for Filipino women with HER2-positive EBC. The model cohorts were Filipino women with HER2-positive EBC who enter the model at the age of 50 years old which is the mean age of patients enrolled in the Department of Health Breast Cancer Medicines Access Program (DOH BCMAP) [36]. We modelled over a lifetime horizon from both publicly-funded healthcare system and societal perspectives, with a discounting rate of 3.5% per year applied to both costs and outcomes. We measured the incremental cost-effectiveness ratio (ICER) as Philippine Peso (PHP) per Life Year (LY) or Quality-adjusted life year (QALY) gained. We applied the current Philippine cost-effectiveness threshold value of PHP 120,000 per QALY gained that was set by the Formulary Executive Council in the Philippines based on the value of one times Gross Domestic Product (GDP) per capita in the Philippines. In the case that trastuzumab was not cost-effective, a threshold analysis was performed to calculate the cost-effective price of trastuzumab therapy. Further, we estimated the likely budget impact of its national coverage for five fiscal years.

### Model overview

**Fig. 1:**
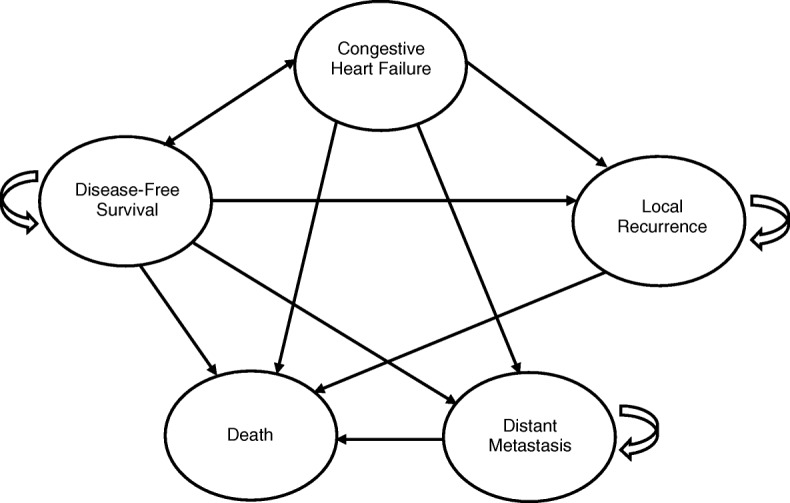
Schematic diagram of the Markov model

Our model structure as illustrated in Fig. 1 consists of five health states - disease-free survival (DFS), congestive heart failure (CHF), local recurrence, distant metastasis, and death. Upon administration of either of the competing interventions, the cohort enters the model at the DFS state. We applied a cycle length of 1 year which was run for 49 cycles to represent lifetime horizon. The model assumed that patients who progressed to CHF could only experience it once as cardiotoxicity from trastuzumab is only an asymptomatic decline in LVEF and is mostly reversible with interruption of trastuzumab or cardiac medication [37]. From CHF state, they have the option to move to DFS, local recurrence, or distant metastasis state.

It was also assumed that the cohort does not have any baseline co-morbidities and the use of hormonal therapies was not considered. Based on the methodological domains and assumptions, a spread sheet model was developed using Microsoft Office Excel 2013 (Microsoft Corp., Redmond, WA) to generate the total lifetime costs incurred and LYs or QALYs gained through cohort simulation.

### Model input parameters

#### Clinical data

The primary data source for the baseline TPs between the health states was obtained from published literatures [31, 38], which were estimated from the four-year follow-up of joint analysis of data from NCCTG N9831 and NSABP B-31 trials on the efficacy and safety trastuzumab plus standard anthracycline-taxane-based CT [39].

We generated the relative treatment effect of trastuzumab by conducting a systematic review and meta-analysis of published trials [9–16] on the efficacy and safety of adjuvant trastuzumab [40]. The benefit of trastuzumab was incorporated in the model as a reduction applied in the risk of developing local recurrence, distant metastasis, and mortality for the intervention model cohort when at DFS state. As for the longest follow-up data available to date showing constant treatment effect over 11 years [9], it was therefore assumed that the efficacy of trastuzumab lasts for 11 years. The cardiotoxic effect was incorporated in the model as increased risk for CHF for the intervention model cohort.

#### Utility data

The utility values associated with the model health states were sourced from a quality of life study among 30 patients each with early and advanced breast cancers at Hanoi Oncology Hospital and Da Nang Oncology Hospital Vietnam [41], which were derived using the EuroQol five-dimension with three-level (EQ-5D-3 L) questionnaires. As the only utility weight study derived from an Asian population in a fellow LMIC setting to date, it was deemed that Vietnam utilities are comparable and applicable to the Philippine setting.

#### Cost data

Direct medical costs (DMC) were comprised of work-up, treatment and monitoring costs related to laboratory and diagnostic tests, procedures, admissions and outpatient visits, and pharmacologic therapy. A pre-constructed costing sheet guided by clinical guidelines [18, 42, 43] was consulted among local experts. We then valuated the final costing items using the government standard case rate values [44] and data from a local costing study [45] for the medical services, procedures, and diagnostics; and, the government drug price reference index [46] and procurement data [47] for all drug costs. The cost per vial of trastuzumab was based on the negotiated price under the DOH BCMAP for the 150 mg-IV-vial preparation amounting to PHP 619,667 for all trastuzumab cycles. The dose calculation was based on a 65 kg-body weight. All costs were expressed in PHP 2017 values and inflation adjustments were applied as necessary by using the Consumer Price Indexes (CPI) [48].

Direct non-medical costs (DNMC) were estimated using the costing values for food and transportation expenses from the Thai Standard Costing Lists for Health Economic Evaluation [49], in the absence of these standard costing values in the Philippines. These costs (in 2009 Thai Baht values) were converted to 2017 PHP values by using the 2009 conversion factors 1 USD = 33.129 Thai Baht [50] and 1 USD = PHP 46.421 [51], then adjusted for inflation using CPI [48]. The utilization of such costs was assumed to incur for every health facility visit involved relevant for the particular health state. The list of all input parameters used in the model is summarized in Table 1.

**Table 1:** Input parameters used in the model and their sampling distribution for the probabilistic sensitivity analysis

Parameter	Mean (SE)	Distribution	Source
Clinical Parameters			
*Baseline Transitional Probabilities*			
DFS ➔ CHF	0.0053 (0.0024)	Beta	Buendia et al., 2013 [31]
DFS ➔ Recurrence	0.0294 (0.0029)	Beta	
DFS ➔ Metastasis	0.0785 (0.0140)	Beta	
DFS ➔ Death	0.0020 (0.0001)	Beta	
CHF ➔ Recurrence	0.0294 (0.0029)	Beta	
CHF ➔ Metastasis	0.0785 (0.0140)	Beta	
CHF ➔ Death	0.1500 (0.0153)	Beta	Dokainish et al., 2017 [52]
Recurrence ➔ Metastasis	0.0785 (0.0140)	Beta	Buendia et al., 2013 [31]
Recurrence ➔ Death	0.2950 (0.2066)	Beta	
Metastasis ➔ Death	0. 0.2950 (0.2066)	Beta	
*Relative treatment efficacy of adjuvant trastuzumab therapy*			
Pooled hazard ratio for DFS	0.65 (0.0825)	Log-Normal	Genuino et al., 2019 [40]
Pooled hazard ratio for OS	0.67 (0.0493)	Log-Normal	
Pooled risk ratio for CHF	3.97 (0.2240)	Log-Normal	
*Epidemiological Data*			
5 – year prevalence of Breast Cancer in the Philippines	64,046 prevalent cases	–	WHO GLOBOCAN, 2012 [8]
Incidence of Breast Cancer in the Philippines	21,057 new cases	–	Estimated based on the 2015 new cases (Philippine Cancer Facts and Estimates, 2015 [2]) adjusted to 2017 values by applying the incidence rate calculated using the total population for 2015 [53] and 2017 [54]
Percentage of HER2-positivity of breast cancer in the Philippines	23.17%	–	DOH Breast Cancer and Control Program, 2013 [7]
Estimated percentage of early-stage HER2-positive breast cancer cases in the Philippines	80%	–	DOH Breast Cancer and Control Program, 2013 [7]
Cost Parameters			
DMC of adjuvant trastuzumab therapy for the intervention cohort *(per patient per treatment course) – Drugs, CT administration, cardiac function assessment*	PHP 1076607 (54929)	Gamma	DOH Philippines -Pharmaceutical Division, 2018 [47] Philippine Health Insurance Corporation, 2013 [44]
DMC of adjuvant CT for the control cohort *(per patient per treatment course) – Drugs, CT administration, cardiac function assessment*	PHP 194900 (9944)	Gamma	
DNMC of adjuvant treatment for intervention cohort	PHP 9432 (481)	Gamma	Riewpaiboon, 2014 [49]
DNMC adjuvant treatment for control cohort	PHP 3494 (178)	Gamma	
DMC at DFS state *- imaging, labs, visits/consultation*	PHP 9493 (484)	Gamma	Wong, 2018 [45]
DNMC at DFS state	PHP 1747 (80)	Gamma	Riewpaiboon, 2014 [49]
DMC at CHF state -*hospital admission, echocardiography, drugs, cardiac monitoring*	PHP 3400 (1602)	Gamma	Philippine Health Insurance Corporation, 2013 [44]
DNMC at CHF state	PHP 1049 [48]	Gamma	Riewpaiboon, 2014 [49]
DMC at Local Recurrence state *(first year) – work-up, radiotherapy, CT drugs, CT administration*	PHP 567156 (3061)	Gamma	DOH Philippines, 2018 [46] Philippine Health Insurance Corporation, 2013 [44]
DNMC at Local Recurrence state *(first year)*	PHP 8166 (1488)	Gamma	Riewpaiboon, 2014 [49]
DMC at Local Recurrence state *(after first year) - CT drugs, CT administration*	PHP 182437 (15513)	Gamma	DOH Philippines, 2018 [46] Philippine Health Insurance Corporation, 2013 [44]
DNMC at Local Recurrence state *(after first year)*	PHP 17817 (178)	Gamma	Riewpaiboon, 2014 [49]
DMC at Local Recurrence state *(after first year) – CT drugs and administration*	PHP 516904 (26373)	Gamma	DOH Philippines, 2018 [46] Philippine Health Insurance Corporation, 2013 [44]
DNMC at Local Recurrence state *(after first year)*	PHP 5939 (273)	Gamma	Riewpaiboon, 2014 [49]
DMC at Distant Metastasis state *(first year) - work-up, radiotherapy, CT drugs, CT administration, palliative care drugs*	PHP 956172 (48784)	Gamma	DOH Philippines, 2018 [46] Philippine Health Insurance Corporation, 2013 [44]
DNMC at Distant Metastasis state *(first year)*	PHP 10131 (465)	Gamma	Riewpaiboon, 2014 [49]
DMC at Distant Metastasis state *(after first year) – CT drugs and administration, palliative care drugs*	PHP 1666816 (59531)	Gamma	DOH Philippines, 2018 [46] Philippine Health Insurance Corporation, 2013 [44]
DNMC at Distant Metastasis state *(after first year)*	PHP 5939 (273)	Gamma	Riewpaiboon, 2014 [49]
Utility Parameters			
DFS	0.8320 (0.0084)	Beta	Ahn et al., 2014 [41]
CHF	0.6700 (272.71)	Beta	
Recurrence	0.8280 (0.0262)	Beta	
Metastasis	0.7620 (0.0262)	Beta	

### Sensitivity analysis

Both one-way and probabilistic sensitivity analyses (PSA) using second order Monte Carlo simulation replicated for 50,000 times were performed to handle parametric uncertainties. We assigned beta distribution for TPs and utility parameters; log-normal distribution for the relative treatment effect; and, gamma distribution for cost parameters. The PSA results were illustrated as incremental cost-effectiveness planes and cost-effectiveness acceptability curves. A discount rate of 0 to 6%, and a treatment efficacy duration of 5 years to 49 years were also applied in the one-way sensitivity analysis.

## Results

### Cost-effectiveness analysis

Based on a probabilistic approach, from the healthcare system perspective, trastuzumab therapy compared to chemotherapy alone was estimated to incur an additional cost of PHP 452,128 with an expected health gain of additional 1.20 LY or 1.00 QALY per patient, resulting in an ICER of PHP 377,009 per LY gained, or PHP 453,505 per QALY gained. From a societal perspective, adopting adjuvant trastuzumab therapy was estimated to cost an additional PHP 457,131 for a similar additional health benefit, resulting to an ICER of PHP 381,405per LY gained, or PHP 458,686 per QALY gained.

The ICERs from both perspectives have consistently shown that adding 1 year of adjuvant trastuzumab to chemotherapy for HER2-positive EBC is not cost-effective in the Philippines as they exceeded the cost-effectiveness threshold by about 3.8 times more. The lifetime costs, LYs and QALYs gained from using trastuzumab therapy versus chemotherapy alone, and the resulting ICERs in the probabilistic analysis from the two perspectives are shown in Table 2.

**Table 2:** Cost-effectiveness results of probabilistic sensitivity analysis

Healthcare system perspective			
	Trastuzumab-CT regimen	CT only regimen	Difference
Total Lifetime Cost (PHP)	4,462,407	4,010,279	452,128
Total LYs gained	11.07	9.87	1.20
Total QALYs gained	8.99	7.99	1.00
ICER (PHP per LY gained)	377,009		
ICER (PHP per QALY gained)	453,505		

### Threshold sensitivity analysis

Results have shown that the cost-effective price of adjuvant trastuzumab therapy per patient is PHP 596,239 from the healthcare system perspective, or PHP 590,314 from a societal perspective. This implies that the current cost of adjuvant trastuzumab therapy overall, including the cost of standard chemotherapy drugs and other supportive medications, chemotherapy administration cost and cardiac function tests (i.e., PHP 1,076,607), needs to be decreased further by about one-half in order to achieve an ICER that will be at least equal to the cost-effectiveness threshold.

### Probabilistic sensitivity analysis

**Fig. 2:**
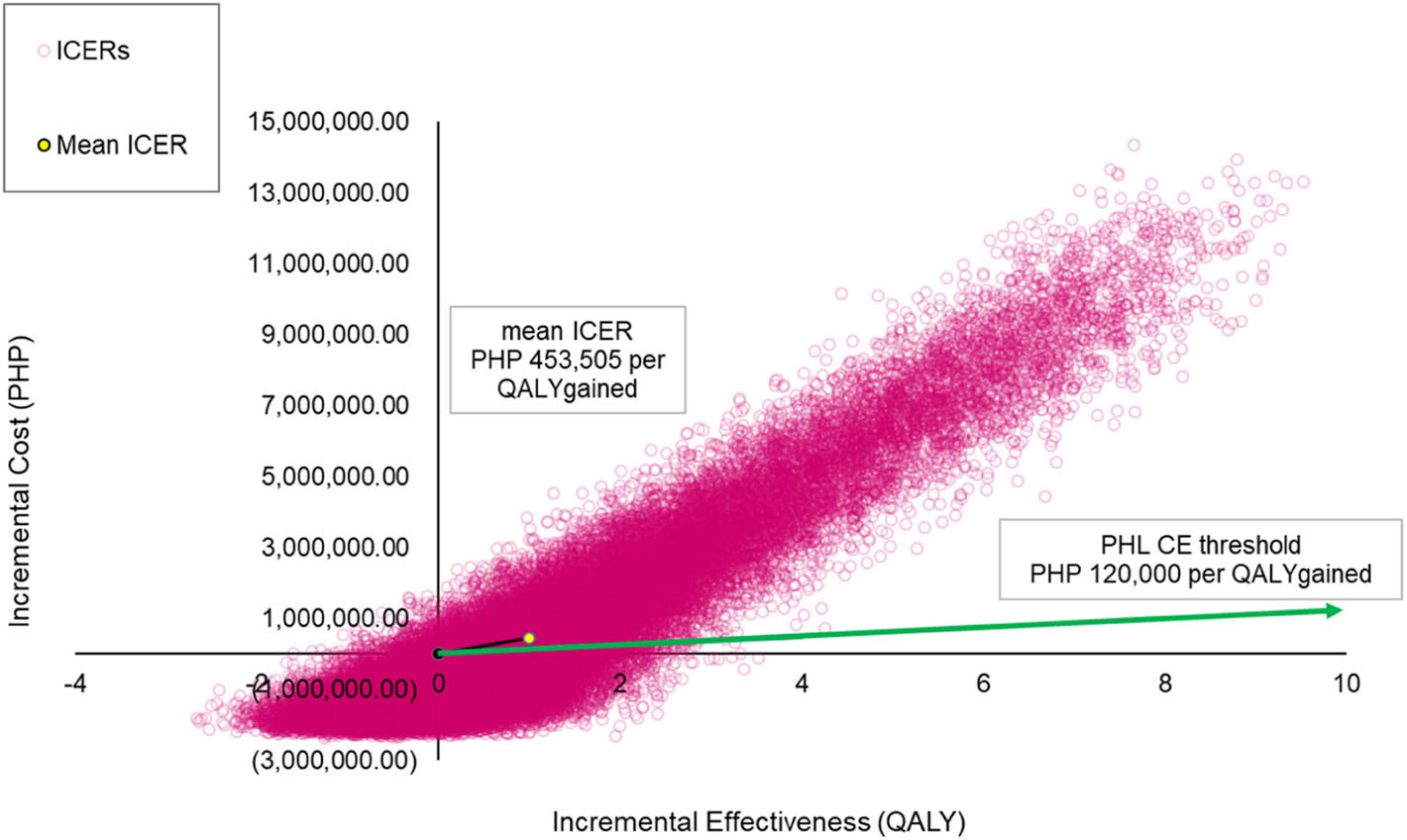
Cost-effectiveness plane from a publicly-funded healthcare system perspective

The results the Monte Carlo simulation with 50,000 replications were plotted in a cost-effectiveness plane, presented in Fig. 2. Majority of the ICER plots appear at the upper-right hand quadrant of the plane extending to form an ellipsoid shape which implies the positive correlation of the incremental cost and the incremental outcomes. The black line represents the resulting mean ICER while the green line represents the cost-effectiveness threshold where estimates below this line are considered cost-effective.

**Fig. 3:**
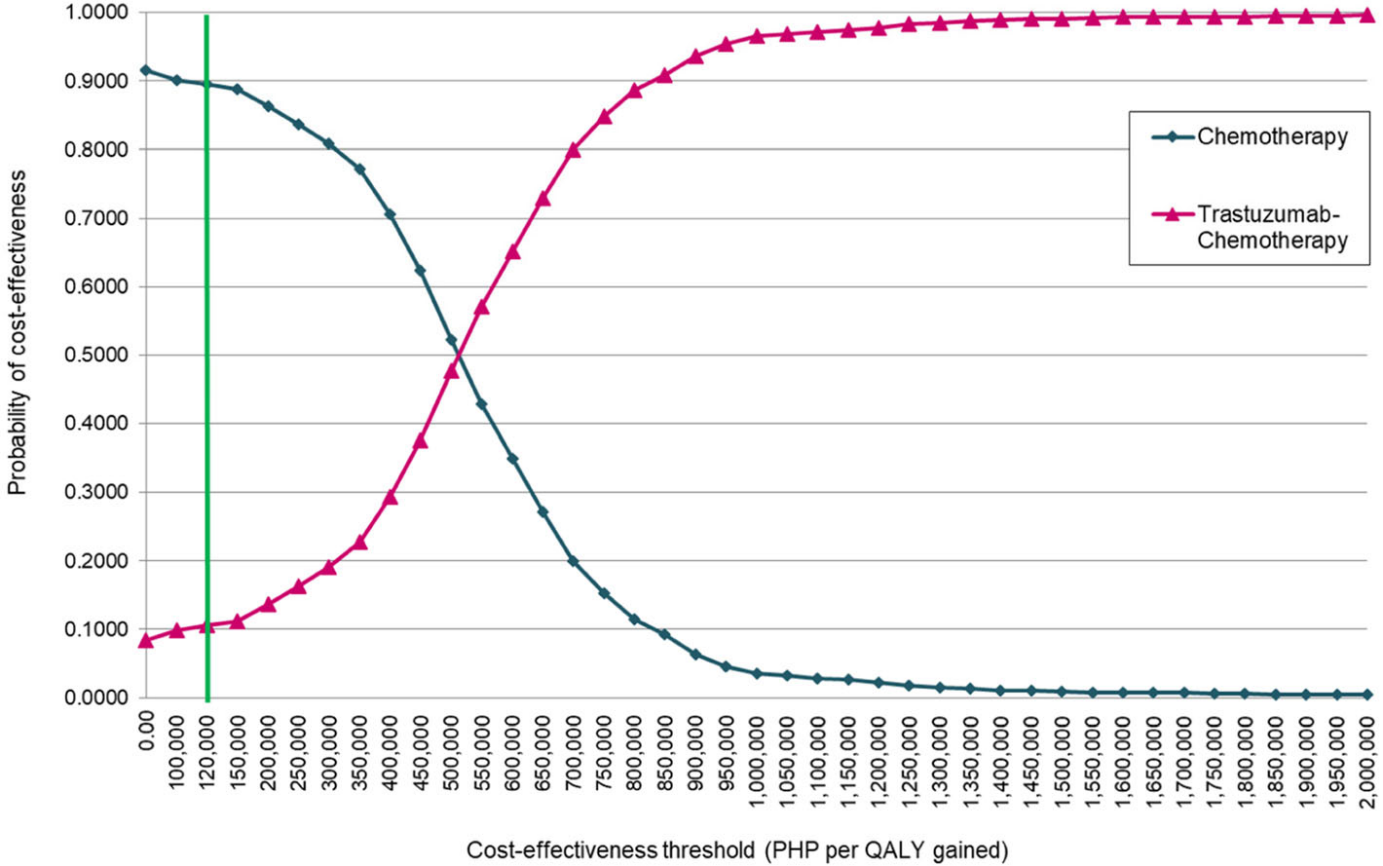
Cost-effectiveness acceptability curve – publicly-funded healthcare system perspective

Figure 3 presents the cost-effectiveness acceptability curve for the healthcare system. Transforming the results of the CE plane to a cost-effectiveness acceptability curve as illustrated in Fig. 3 demonstrates that at the cost-effectiveness threshold of PHP 120,000 per QALY gained (green line), the probability of cost-effectiveness of adjuvant trastuzumab therapy is 10%, while that for the chemotherapy alone regimen is 90%, under both perspectives. The current cost-effectiveness threshold has to increase by about four times more (i.e., PHP 500,000 per QALY gained) in order for adjuvant trastuzumab to reach at least 48% probability of cost-effectiveness from the publicly-funded healthcare system perspective, or 51% probability of cost-effectiveness from the societal perspective. The trastuzumab curve only started to rise steeply as the threshold increases at PHP 650,000 per QALY gained.

### Deterministic sensitivity analysis

**Fig. 4:**
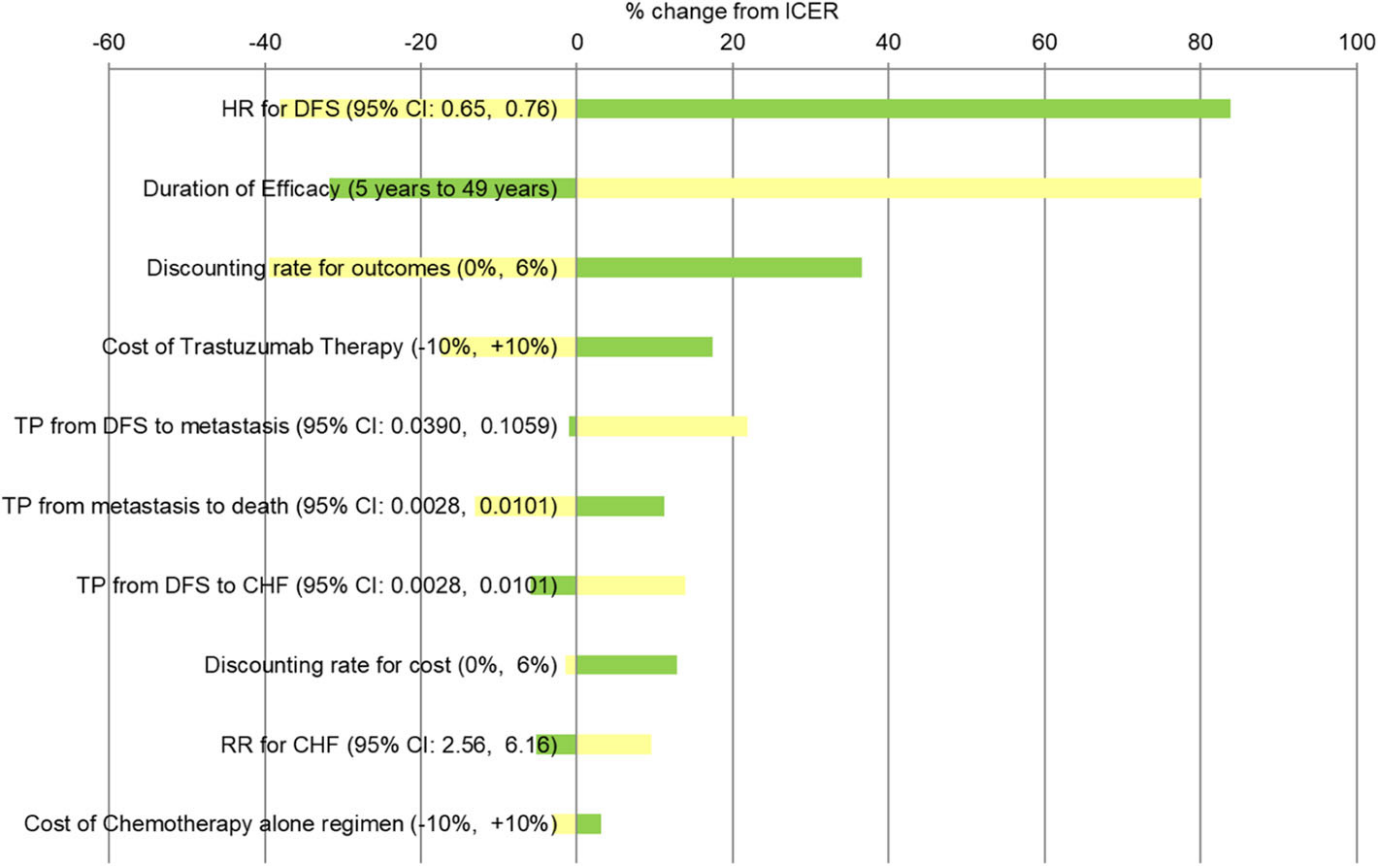
One-way sensitivity analysis from a publicly-funded healthcare system perspective

The results of the one-way sensitivity analyses are presented in Fig. 4. Among all input parameters varied under both perspectives, the ICER results were most sensitive to variations in the hazard ratio for DFS, duration of efficacy of trastuzumab, discounting rate for outcomes, cost of trastuzumab therapy, and TP from DFS to metastasis state. On the other hand, the model estimates were negligibly sensitive with respect to variations in the DMC and DNMC for all health states, and all utilities at the different health states. The yellow bars show the effect on the ICER of applying the lower limit of the specific parameter, while the green bars show the effect on the ICER of applying the upper limit of the specific parameter.

**Fig. 5:**
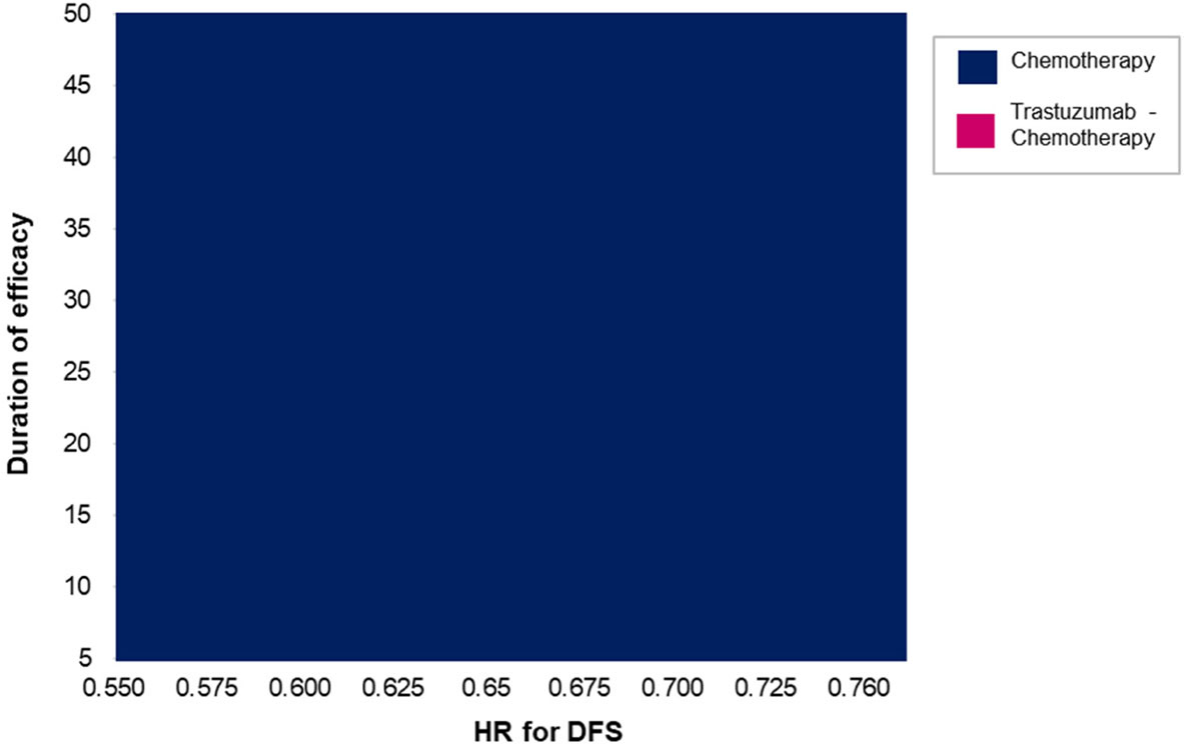
Two-way sensitivity analysis from a publicly-funded healthcare system perspective

We likewise explored the impact on the cost-effectiveness of adjuvant trastuzumab therapy of simultaneous changes in the values of the HR of DFS and the duration of efficacy. The two-way sensitivity analysis as illustrated in Fig. 5 shows that over the plausible range of the said parameters, standard chemotherapy remained the cost-effective approach.

### Budget impact analysis

The estimated number of prevalent cases of HER2-positive EBC in the Philippines is 11,872 which was calculated based on the estimated proportion of early-stage cases (i.e., 80% [7]) among all HER2-positive patients which is about 23.17% (i.e., HER2-positivity rate in the Philippines [7]) of the total breast cancer prevalent cases in the country [8]. The same calculation was performed for the generation of the estimated new cases of 3903 by calculating the proportions of early-stage HER2-positive cases from the total breast cancer new cases in the Philippines [2] adjusted to 2017 value.

**Fig. 6:**
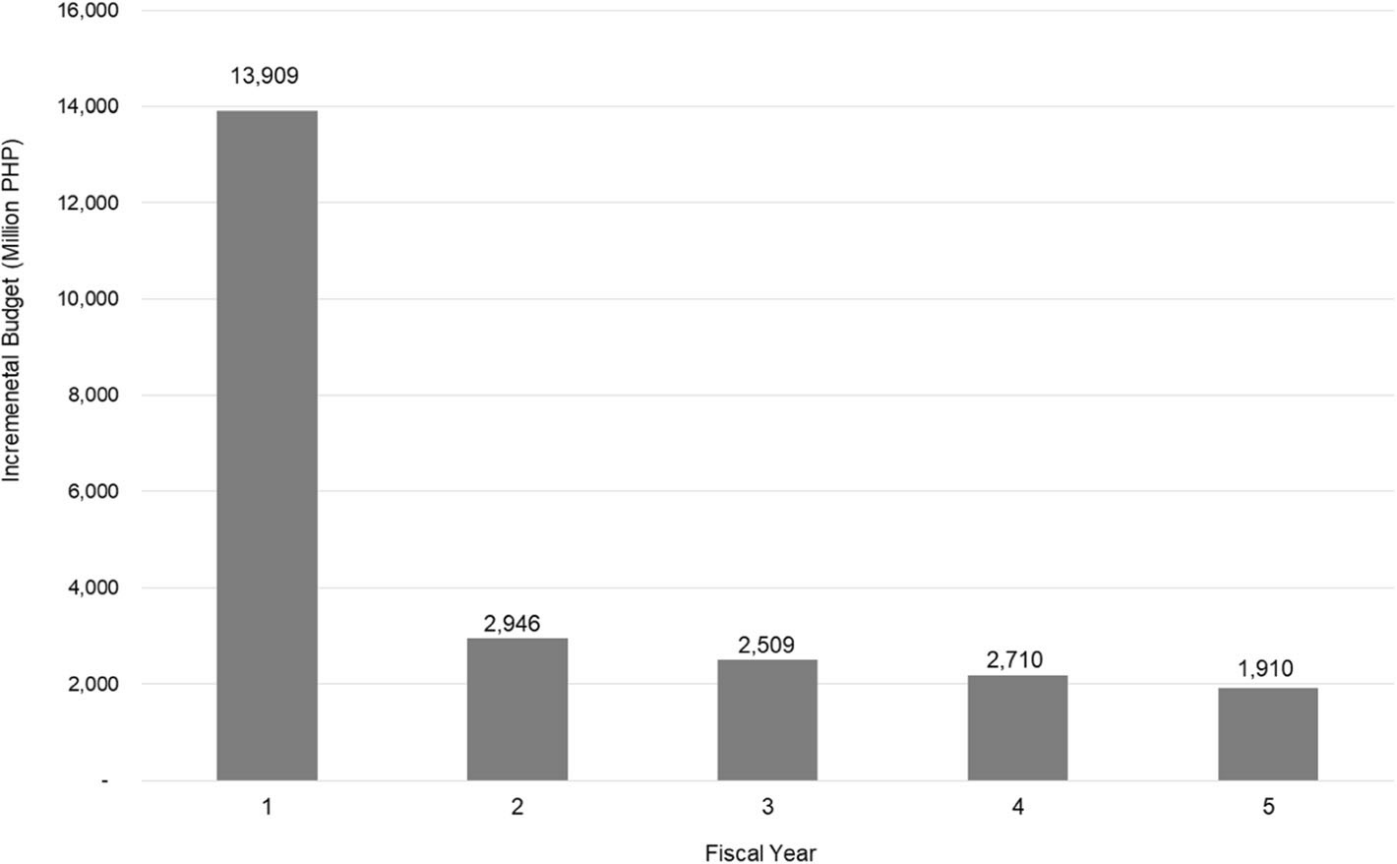
Five-year governmental budget impact

The total budget for implementing adjuvant trastuzumab therapy to cover all estimated prevalent and new cases of HER2-positive EBC patients in the Philippines will demand high health budget as it will incur PHP 16,983 million per cohort in year one alone, plus more than PHP 5200 million annually per cohort on the next four fiscal years. On the contrary, implementing chemotherapy alone to cover the same number of prevalent and new cases will incur a total cost of PHP 3075 million in year one, and more than PHP 2300 million annually on the next four fiscal years. The incremental budget, therefore, is about PHP 13,909 million in year one alone, plus about PHP 2000 to 3000 million annually on the next four fiscal years, as presented in Fig. 6.

## Discussion

To our knowledge, this is the first published study in Philippines and among LMICs to evaluate the health and economic impact of adjuvant trastuzumab therapy for HER2-positive EBC. The study is intended to guide coverage decisions on such effective but high cost therapy under a low-resource setting. It is believed that this economic evaluation faithfully considered and represented all the best available evidence appropriate to a developing country context by conducting a systematic review and meta-analysis to estimate the relative treatment effect.

In this model, we project that shifting to 1 year of adjuvant trastuzumab therapy in addition to chemotherapy for HER2-positive EBC, at its current cost, shall incur additional cost to the government of PHP 453,505 or USD 9084, or the society with additional PHP 458,686or USD 9188 (2017 Exchange Rate: 1 USD = PHP 49.9230 [51],) for every unit of QALY gained. Exceeding the cost-effectiveness threshold by 3.8 times more, our findings suggest that such therapy is not cost-effective in the Philippines with 10% of being cost-effective at the threshold compared to chemotherapy alone at 90%. Its value for money will improve if its current therapy cost will reduce by one-half. Apart from cost-effectiveness, the decision makers will also have to consider the affordability of trastuzumab coverage. The total acquisition cost for trastuzumab drug alone for a national coverage implementation will cost about PHP 7.36 billion – an amount that significantly exceeds the usual annual budget for the procurement of various breast cancer medicines covered under the DOH BCMAP (i.e., ranging from PHP 39 to 92 million from year 2009 to 2017) and can consume the chunk of the DOH budget allocation for the procurement of all drugs under the national health programs (i.e., PHP 9.87 billion for 2018) [55, 56].

Our generalizations are comparable with majority of previous EEs from upper-middle-income countries (UMICs) [31–33] which similarly concluded that trastuzumab in their settings is not cost-effective. In Iran [33], the ICER is about USD 174,901 per QALY gained which is 17 times more than its cost-effectiveness threshold. The ICERs range from about USD 63,036 to 153,554 in Latin American countries [31, 32], similarly exceeding their cost-effectiveness threshold by 6 to 15 times more. On the contrary, the CUA studies from high-income countries (HICs) [20–24, 26–30, 34, 35] concluded that trastuzumab is cost-effective in their settings with their ICERs ranging from USD 11,334 to 78,929 per QALY gained. Trastuzumab though was found to be cost-effective in the analyses from two UMICs specifically in China by Chen et al., 2009 [25] and in Thailand by Kongsakon et al., 2018 [38], with ICERs at USD 9976 per QALY gained and USD 6527 per QALY gained, respectively. Such relatively lower ICER and opposing conclusion compared to the earlier mentioned findings from majority of analysis from UMICs [31–33] may be explained by several factors. Chen et al., 2009 applied a relatively lower hazard ratio (i.e., 0.54) versus those used by the studies in Iran (i.e., 0.64) and Latin America (i.e., 0.59). Second, it applied a 5-year efficacy duration with decreasing efficacy in a stepwise function, compared to the studies in Iran and Latin America which applied a 5-year duration of efficacy with zero applied benefit onwards. Kongsakon et al., 2018 [38] did not incorporate cardiac events in their modelling analysis which may have resulted to an underestimated ICER.

Considering ICER values alone, our ICERs (~ USD 9084 per QALY gained) were in fact lower than the ICER range from UMICs (USD 63,036 to 174,901 per QALY gained) which concluded for non-cost-effectiveness [31–33], the ICER range from HICs (USD 11,334 to 78,929 per QALY gained) which concluded for cost-effectiveness [20–24, 26–30, 34, 35]. Two factors in this analysis may have contributed to our relatively low ICER: First is the longer duration of trastuzumab efficacy applied in this modelling (i.e., 11 years) versus most of the previous studies (i.e., 5 years) which was based on the currently available follow-up data during the time of those EEs; and, second is the lower cost of trastuzumab therapy in our analysis (USD 12,412) versus other studies (USD 35,349 to 137,677). Our low ICER, however, did not automatically translate to favourable cost-effectiveness findings because of the relatively low Philippine cost-effectiveness threshold at USD 2404 per QALY gained only, compared to the higher thresholds of UMICs (~USD 5000 to 15,000 per QALY gained) and HICs (USD 37,920 to 354,555).

Our results were specifically sensitive to the following parameters i.e., the relative treatment efficacy which underscores the significance of estimating the effectiveness from real-world data where a less favourable treatment effect in the actual clinical practice setting may be possibly observed compared to follow-up studies under controlled environment; the duration of clinical efficacy which supports the significance of longer follow-up data on trastuzumab to define its real duration of benefit, and therefore its value for money; and, the cost of trastuzumab therapy which emphasizes the need to consider schemes to bring down its incurred cost to the government in order to achieve cost-effectiveness. Nevertheless, the model and its overall findings proved to be relatively robust, showing minimal sensitivity with variations in the majority of the parameters.

There were several limitations in this analysis. First, we referred to the Vietnamese utility data for breast cancer in the absence of such data in the Philippines. While there may be differences between Filipino and Vietnamese patients on the valuation of health states because of numerous factors (e.g., cultural differences affecting disease perception, social support, health system structure), this approach was deemed acceptable because of a comparable Asian context and economic status. Second, we used the Thai cost values for the DNMCs in the societal perspective analysis due to data unavailability. As the only available and accessible standard costing menu from a similar Asian setting, using the Thai cost values was likewise deemed acceptable. The recommended study perspective anyway in the Philippine methods guide for economic evaluation is publicly-funded healthcare system which, in our analysis, used purely local costing data. Lastly, the estimation of baseline TPs were obtained from published data, not local data. Hence, it is recommended in future analysis to re-run the modelling using local TPs, health utilities and DNMC values. Exploring the role of subgroups (i.e., hormone-receptor and nodal status) on the value for money of trastuzumab is likewise proposed.

## Conclusion

In conclusion, this CUA suggests that 1 year of adjuvant trastuzumab therapy, at its current cost, in addition to the standard chemotherapy for HER2-positive EBC, might not be cost-effective and unaffordable in the Philippines. The government should consider schemes to lower down the cost to improve its value for money such as price negotiation, facilitation of the entry of cheaper biosimilar products, or targeted coverage (for the worst prognosis subtype or high risk subgroups), ultimately towards sustainable access to trastuzumab.

Our findings are designed to aid policy decisions of a resource-constrained setting on the coverage of such effective but high cost therapy. As this research is the first published study among LMICs to evaluate the health and economic impact of adjuvant trastuzumab therapy for HER2-positive EBC, our findings may guide decision-makers from comparable low resource settings with low capacity to conduct economic evaluations who are also facing a similar policy and research question.

## Data Availability

The patient data that support the findings of this study are available from the DOH and PCS but restrictions apply to the availability of these data, which were used under license for the current study, and so are not publicly available. Data are however available from the authors upon reasonable request and with permission of the DOH and PCS.

## Abbreviations

CHF: Congestive heart failure
CPI: Consumer price index
CT: Chemotherapy
CUA: Cost-utility analysis
DMC: Direct medical cost
DNMC: Direct non-medical cost
DOH BCMAP: Department of Heath Breast Cancer Medicine Access Program
DOH: Department of Heath
EBC: Early stage breast cancer
EE: Economic evaluation
HER2: Human epidermal growth factor receptor 2
HIC: High income country
HR: Hazard ratio
ICER: Incremental cost-effectiveness ratio
LMIC: Lower-middle income country
LVEF decline: Left ventricular ejection fraction decline
LY: Life year
OS: Overall survival
PCS: Philippine Cancer Society
PHL CE threshold: Philippine cost-effectiveness threshold
PHP: Philippine Peso
PNF: Philippine National Formulary
PSA: Probabilistic sensitivity analysis
QALY: Quality adjusted life year
RR: Risk ratio
TP: Transition probability
UMIC: Upper-middle income country
WHO: World Health Organization

## References

[1] Cancer WHO-IAfRo. GLOBOCAN 2012: estimated cancer incidence, mortality and prevalence worldwide in 2012 [web page]: World Health Organization; 2012. Available from: http://globocan.iarc.fr/Pages/fact_sheets_cancer.aspx. Accessed 15 Dec 2018.

[2] Laudico AVM-LM, Medina V, Mapua C, Valenzuela F, Pukkala E (2015). Philippine cancer facts and estimates.

[3] Wolff ACHM, Hicks DG, Dowsett M, McShane LM, Allison KH, Allred DC, Bartlett JM, Bilous M, Fitzgibbons P, Hanna W, Jenkins RB, Mangu PB, Paik S, Perez EA, Press MF, Spears PA, Vance GH, Viale G, Hayes DF (2013). Recommendations for human epidermal growth factor receptor 2 testing in breast cancer: American Society of Clinical Oncology/College of American Pathologists clinical practice guideline update. J Clin Oncol.

[4] Gajria D, Chandarlapaty S (2011). HER2-amplified breast cancer: mechanisms of trastuzumab resistance and novel targeted therapies. Expert Rev Anticancer Ther.

[5] Kwan MLKL, Weltzien E, Maring B, Kutner SE, Fulton RS, Lee MM, Ambrosone CB, Caan BJ (2009). Epidemiology of breast cancer subtypes in two prospective cohort studies of breast cancer survivors. Breast Cancer Res.

[6] Parise CABK, Brown MM, Caggiano V (2009). Breast cancer subtypes as defined by the estrogen receptor (ER), progesterone receptor (PR), and the human epidermal growth factor receptor 2 (HER2) among women with invasive breast cancer in California, 1999-2004. Breast J.

[7] Philippines DoH-NBCCP (2013). National breast cancer control program - HER2 testing census. In: Bureau DoH-DPaC, editor.

[8] Organization WH (2012). GLOBOCAN 2012: estimated cancer incidence, mortalitu and prevalence worldwide in 2012 IARC.

[9] Cameron DP-GM, Gelber RD, Procter M, Goldhirsch A, de Azambuja E (2017). 11 years’ follow-up of trastuzumab after adjuvant chemotherapy in HER2-positive early breast cancer: final analysis of the HERceptin Adjuvant (HERA) trial. Lancet.

[10] Perez EARE, Suman VJ, Jeong JH, Sledge G, Geyer CE, Martino S, Rastogi P, Gralow J, Swain SM, Winer EP, Colon-Otero G, Davidson NE, Mamounas E, Zujewski JA, Wolmark N (2014). Trastuzumab plus adjuvant chemotherapy for human epidermal growth factor receptor 2–positive breast cancer: planned joint analysis of overall survival from NSABP B-31 and NCCTG N983. J Clin Oncol.

[11] Romond EHJJ, Rastogi P, Swain SM, Geyer CE, Ewer MS, Rathi V, Fehrenbacher L, Brufsky A, Azar CA, Flynn PJ, Zapas JL, Polikoff J, Gross HM, Biggs DD, Atkins JN, Tan-Chiu E, Zheng P, Yothers G, Mamounas EP, Wolmark N (2012). Seven-year follow-up assessment of cardiac function in NSABP B-31, a randomized trial comparing doxorubicin and cyclophosphamide followed by paclitaxel (ACP) with ACP plus trastuzumab as adjuvant therapy for patients with node-positive, human epidermal growth factor receptor 2–positive breast cancer. J Clin Oncol.

[12] Advani PPBK, Dockter TJ, Colon-Otero G, Perez EA (2016). Long term cardiac safety analysis of NCCTG N9831 (alliance) adjuvant trastuzumab trial. J Clin Oncol.

[13] Slamon DEW, Robert N, Pienkowski T, Martin M, Press M, Mackey J, Glaspy J, Chan A, Pawlicki M, Pinter T, Valero V, Liu MC, Sauter G, von Minckwitz G, Visco F, Bee V, Buyse M, Bendahmane B, Tabah-Fisch I, Lindsay MA, Riva A (2011). Adjuvant trastuzumab in HER2-positive breast cancer. N Engl J Med.

[14] Joensuu HBP, Kataja V, Alanko T, Kokko R, Asola R, Utriainen T, Turpeenniemi-Hujanen T, Jyrkkiö S, Möykkynen K, Helle L, Ingalsuo S, Pajunen M, Huusko M, Salminen T, Auvinen P, Leinonen H, Leinonen M, Isola J, Kellokumpu-Lehtinen PL (2009). Fluorouacil, epirubicin and cyclophosphamide with either docetaxel or vinorelbine, with or without trastuzumab, as adjuvant treatments of breast cancer: final results of the FinHer trial. J Clin Oncol.

[15] Spielmann MRH, Delozier T, Canon JL, Romieu G, Bourgeois H, Extra JM, Serin D, Kerbrat P, Machiels JP, Lortholary A, Orfeuvre H, Campone M, Hardy-Bessard AC, Coudert B, Maerevoet M, Piot G, Kramar A, Martin AL, Penault-Llorca F (2009). Trastuzumab for patients with axillary-node-positive breast cancer: results of the FNCLCC-PACS 04 trial. J Clin Oncol.

[16] Joensuu HK-LP, Huovinen R, Jukkola-Vuorinen A, Tanner M, Kokko R, Ahlgren J, Auvinen P, Saarni O, Helle L, Villman K, Nyandoto P, Nilsson G, Leinonen M, Kataja V, Bono P, Lindman H (2014). Outcome of patients with HER2-positive breast cancer treated with or without adjuvant trastuzumab in the Finland Capecitabine Trial (FinXX). Acta Oncol.

[17] Senkus EKS, Ohno S, Penault-Llorca F, Poortmans P, Rutgers E, Zackrisson S, Cardoso F (2015). Primary breast cancer: ESMO Clinical Practice Guidelines for diagnosis, treatment and follow-up. Ann Oncol.

[18] Network. NCC (2017). Guidelines clinical practice guidelines in oncology- breast cancer version 2.2017.

[19] Force PSoMO-CPT (2015). Clinical pathways for the medical management of the top 10 solid malignant tumors in the Philippines Philippine Society of Medical Oncology.

[20] Dedes KJST, Imesch P, Fedier A, Fehr MK, Fink D (2007). Cost-effectiveness of trastuzumab in the adjuvant treatment of early breast cancer: a model-based analysis of the HERA and FinHer trial. Ann Oncol.

[21] Garrison LP, Lubeck D, Lalla D, Paton V, Dueck A, Perez EA (2007). Cost-effectiveness analysis of trastuzumab in the adjuvant setting for treatment of HER2-positive breast cancer. Cancer.

[22] Kurian AWTR, Gaw AF, Arai S, Ortiz R, Garber AM (2007). A cost-effectiveness analysis of adjuvant trastuzumab regimens in early HER2/neu-positive breast cancer. J Clin Oncol.

[23] Liberato NLMM, Barosi G (2007). Cost effectiveness of adjuvant trastuzumab in human epidermal growth factor receptor 2-positive breast cancer. J Clin Oncol.

[24] Shiroiwa TFT, Shimozuma K, Ohashi Y, Tsutani K (2008). The model-based cost-effectiveness analysis of 1-year adjuvant trastuzumab treatment: based on 2-year follow-up HERA trial data. Breast Cancer Res Treat.

[25] Chen WJZ, Shao Z, Sun Q, Shen K (2009). An economic evaluation of adjuvant trastuzumab therapy in HER2-positive early breast cancer. Value Health.

[26] Skedgel CRD, Younis T (2009). The cost-utility of sequential adjuvant trastuzumab in women with Her2/Neu-positive breast cancer: an analysis based on updated results from the HERA trial. Value Health.

[27] Van Vlaenderen ICJ, Cocquyt V, Jerusalem G, Machiels JP, Neven P, Nechelput M, Delabaye I, Gyldmark M, Annemans L (2009). Trastuzumab treatment of early stage breast cancer is cost-effective from the perspective of the Belgian health care authorities. Acta Clin Belg.

[28] Macedo AMI, Andrade S, Cirrincione A, Ray J (2010). Cost-effectiveness of trastuzumab in the treatment of early stages breast cancer patients, in Portugal. Acta Medica Port.

[29] Hall PSHC, McCabe C, Oluboyede Y, Round J, Cameron DA (2011). Updated cost-effectiveness analysis of trastuzumab for early breast cancer: a UK perspective considering duration of benefit, long-term toxicity and pattern of recurrence. Pharmacoeconomics.

[30] Hedden LORS, Lohrisch C, Chia S, Speers C, Kovacic L, Taylor S, Peacock S (2012). Assessing the real-world cost-effectiveness of adjuvant trastuzumab in HER-2/neu positive breast cancer. Oncologist.

[31] Buendía JAVC, Pichón-Rivière A (2013). An economic evaluation of trastuzumab as adjuvant treatment of early HER2-positive breast cancer patients in Colombia. Biomedica.

[32] Pichon-Riviere AGO, Augustovski F, Vallejos C, Huayanay L, Bueno Mdel P, Rodriguez A, de Andrade CJ, Buendía JA, Drummond M (2015). Implications of global pricing policies on access to innovative drugs: the case of trastuzumab in Seven Latin American Countries. Int J Technol Assess Health Care.

[33] Aboutorabi AHM, Ghaderi H, Salehi M, Ghiasipour M (2015). Cost-effectiveness analysis of trastuzumab in the adjuvant treatment for early breast cancer. Global J Health Sci.

[34] Lang HCCH, Chiou TJ, Chan AL (2016). The real-world cost-effectiveness of adjuvant trastuzumab in HER-2/neu-positive early breast cancer in Taiwan. J Med Econ.

[35] Leung WKG, Nair N, Blakely T (2016). Adjuvant trastuzumab in HER2-positive early breast cancer by age and hormone receptor status: a cost-utility analysis. PLoS Med.

[36] Society PC (2018). DOH breast cancer medicine access program (BCMAP) follow-up data of non-trastuzumab HER2+ breast cancer patients. In: Society PC, editor. Manila.

[37] Tanaka S, Ikari A, Nitta T, Horiuchi T. Long-term irreversible trastuzumab-induced cardiotoxicity for metastatic breast cancer in a patient without cardiac risk factors. Oxf Med Case Reports. 2017;2017(7). 10.1093/omcr/omx038. 10.1093/omcr/omx038 PMC5499211 28694974

[38] Kongbutr R, Koontreer S, Traianat N, Vatakapan C (2012). Economic evaluation of trastuzumab for treatment of breast cancer in Thailand.

[39] Pérez EARE, Suman VJ, Jeong JH, Davidson NE, Geyer CE (2011). Four-year follow-up of trastuzumab plus adjuvant chemotherapy for operable human epidermal growth factor receptor 2-positive breast cancer: joint analysis of data from NCCTG N9831 and NSABP B-31. J Clin Oncol.

[40] Genuino AJM, Chaikledkaew U, Ong DT, Reungwetwattana T, Thakkinstian A (2018). Adjuvant trastuzumab regimen for HER2-positive early-stage breast cancer: a systematic review and meta-analysis.

[41] Anh NQNHV, Ha NT, Anh N, Thuy H, Hanh NTM (2014). Assessment of the quality of life of breast and cervical cancer in Viet Nam in 2014.

[42] Group DBCCPGTW (2014). Clinical practice guidelines for stages 0-IIIB breast cancer at first diagnosis. In: Division D-P, editor. 1 ed.

[43] Force PSoMO-CPT (2015). Clinical pathways for the medical management of the top 10 solid malignant tumors in the Philippines 2015. Manila.

[44] Corporation PHI (2013). PHIC circular 0035 s.2013 - implementing guidelines on medical and procedure case rates.

[45] Wong JQ (2018). Facility-based intervention costing for the 48 highest burden diseases in the guaranteed health benefits package of the Department of Health and the Philippine Health Insurance Corporation. In: Technology DoSa, editor.

[46] Philippines DoH (2018). The Philippine drug price reference index 2017. In: Philippines DoH, editor.

[47] Division DHP-P (2018). Breast cancer medicines access program procurement data 2011–2017. In: Division DoHP-P, editor.

[48] Group TWB (2018). Consumer price index (1960–2017) - International Monetary Fund, International Financial Statistics and data files: The World Bank Group.

[49] Riewpaiboon A (2014). Standard cost lists for health economic evaluation in Thailand. Med Assoc Thai.

[50] Fund IM (2014). Representative exchange rates for selected currencies for December 2014: International Monetary Fund.

[51] Philippines BSnPCBot (2018). Philippine peso per US Dollar exchange rates - daily, monthly, annual Manila, Philippines.

[52] Dokainish HTK, Zhu J, Roy A, AlHabib KF, ElSayed A, Palileo-Villaneuva L, Lopez-Jaramillo P, Karaye K, Yusoff K, Orlandini A, Sliwa K, Mondo C, Lanas F, Prabhakaran D, Badr A, Elmaghawry M, Damasceno A, Tibazarwa K, Belley-Cote E, Balasubramanian K, Islam S, Yacoub MH, Huffman MD, Harkness K, Grinvalds A, McKelvie R, Bangdiwala SI, Yusuf S, INTER-CHF Investigators (2017). Global mortality variations in patients with heart failure: results from the International Congestive Heart Failure (INTER-CHF) prospective cohort study. Lancet Glob Health.

[53] Authority PS (2016). Highlights of the Philippine population 2015 census of population Manila.

[54] Bank TW (2018). Total population - all countries and economies.

[55] Division DP-P (2018). Total spending for DOH BCMAP In: Division P, editor. Manila.

[56] Bureau DP-DPaC (2017). Work and financial plan 2017 - breast cancer drugs. In: Bureau DPaC, editor. Manila.

